# Designing and implementing two facilitation interventions within the ‘Facilitating Implementation of Research Evidence (FIRE)’ study: a qualitative analysis from an external facilitators’ perspective

**DOI:** 10.1186/s13012-018-0812-z

**Published:** 2018-11-16

**Authors:** Gill Harvey, Brendan McCormack, Alison Kitson, Elizabeth Lynch, Angie Titchen

**Affiliations:** 1 Adelaide Nursing School, University of Adelaide, Adelaide, Australia; 2 Division of Nursing, Queen Margaret University , Edinburgh, Scotland; 3 College of Nursing and Health Sciences, Flinders University, Adelaide, Australia; 4 Formerly Institute of Nursing and Health Research, University of Ulster, Coleraine, Northern Ireland; 5 Formerly Knowledge Centre for Evidence-based Practice, Fontys University of Applied Sciences, Eindhoven, The Netherlands

**Keywords:** Facilitation, Internal-external facilitators, PARIHS, Fidelity, Adaptation

## Abstract

**Background:**

The ‘Facilitating Implementation of Research Evidence’ study found no significant differences between sites that received two types of facilitation support and those that did not on the primary outcome of documented compliance with guideline recommendations. Process evaluation highlighted factors that influenced local, internal facilitators’ ability to enact the roles as envisaged. In this paper, the external facilitators responsible for designing and delivering the two types of facilitation intervention analyse why the interventions proved difficult to implement as expected, including the challenge of balancing fidelity and adaptation.

**Methods:**

Qualitative data sources included notes from monthly internal-external facilitator teleconference meetings, from closing events for the two facilitation interventions and summary data analyses from repeated interviews with 16 internal facilitators. Deductive and inductive data analysis was led by an independent researcher to evaluate how facilitation in practice compared to the logic pathways designed to guide fidelity in the delivery of the interventions.

**Results:**

The planned facilitation interventions did not work as predicted. Difficulties were encountered in each of the five elements of the logic pathway: recruitment and selection of appropriate internal facilitators, preparation for the role, ability to apply facilitation knowledge and skills at a local level, support and mentorship from external facilitators via monthly teleconferences, working collaboratively and enabling colleagues to implement guideline recommendations. Moreover, problems were cumulative and created tensions for the external facilitators in terms of balancing the logic pathway with a more real-world, flexible and iterative approach to facilitation.

**Conclusion:**

Evaluating an intervention that is fluid and dynamic within the methodology of a randomised controlled trial is complex and challenging. At a practical level, relational aspects of facilitation are critically important. It is essential to recruit and retain individuals with the appropriate set of skills and characteristics, explicit support from managerial leaders and accessible mentorship from more experienced facilitators. At a methodological level, there is a need for attention to the balance between fidelity and adaptation of interventions. For future studies, we suggest a theoretical approach to fidelity, with a focus on mechanisms, informed by prospective use of process evaluation data and more detailed investigation of the context-facilitation dynamic.

## Background

The ‘Facilitating Implementation of Research Evidence (FIRE)’ study set out to compare two different facilitation approaches against standard dissemination of clinical guideline recommendations [1]. Both approaches comprised facilitator roles and facilitation processes, but were underpinned by different theories, which determined the focus of the role and corresponding skills and knowledge requirements. In both approaches, a model of external-internal facilitation was employed. Separate papers describe the outcome and process findings. There were no significant differences between the three study arms (control and two facilitation types) on the primary outcome of documented compliance with continence guideline recommendations [2]. The realist process evaluation suggested an interplay between mechanisms relating to the alignment and fit of the facilitation intervention with the internal facilitator (IF) and their work setting, prioritisation of the topic of continence and engagement with the intervention, which, in combination influenced the IFs’ ability to learn over time and enact the role as envisaged [3]. In both types of facilitation, there were examples where individuals in the IF role did and did not enact the role as intended. In turn, this influenced their ability to effect changes in processes and outcomes of care.

Reflecting on the findings of the FIRE study and our experiences as external facilitators (EFs) led to us conducting a more detailed, retrospective analysis of the process of implementing the two facilitation interventions in an attempt to further understand the observed variations. Specifically we undertook to question: Why did the facilitation interventions, as articulated in the study protocol, prove difficult to implement in practice? What issues arose in relation to balancing fidelity and adaptation? What lessons were learned that could be beneficial to inform similar research in the future?

The paper commences with a description of the interventions labelled type A and type B facilitation and strategies employed by EFs to prepare, mentor and support IFs. This includes a description of the ‘logic pathway’ [4] of manualised facilitation interventions, which was developed from the study protocol to guide fidelity. This is followed by an overview of study methods relevant to this paper. Results are presented in relation to the pathway of how the interventions were expected to work and what actually happened in reality. This frames the discussion of factors influencing the enactment of facilitation roles and processes and what we would do differently with the benefit of hindsight.

### The facilitation interventions

Facilitation is one of three constructs in the PARIHS framework, alongside evidence and context [5–7]. It represents the active ingredient of implementation, with individuals defined as facilitators taking on a change agency role to identify elements of evidence and context that might influence implementation and then utilising appropriate facilitation methods and processes to enable the implementation process.

Facilitation is underpinned by a range of theoretical perspectives and influences, including education, counselling, critical social science, management studies and community development [8–12]. The way in which the role, and accompanying facilitation method, is interpreted depends upon the underlying theoretical perspective, and this has implications for preparing and developing individuals to take on the role.

**Fig. 1:**
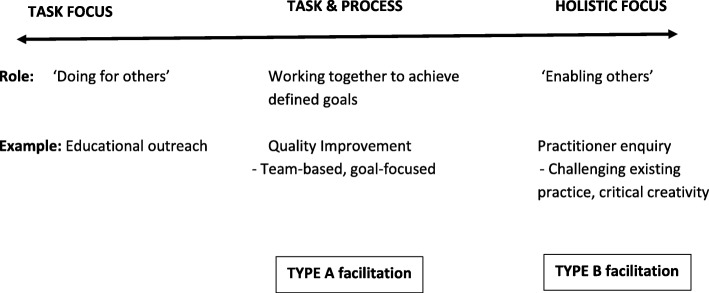
Facilitation continuum

In the development of PARIHS, a concept analysis of facilitation was conducted [13]. Reflecting the multiple theoretical influences, the concept was represented along a continuum, ranging from a largely task and project-focused concern to a person-centred, enabling and emancipatory approach. At a conceptual level, the dynamic interplay between evidence and context indicated the need for flexibility, with facilitators having the ability to move along the continuum depending on the needs of the specific situation. In practice, the facilitation approaches employed by members of the PARIHS group reflected two main traditions of quality improvement and practice development [5], which could be positioned at different points from the mid- to right-hand side of the facilitation continuum (see Fig. 1). This formed the starting point for designing the interventions to be tested in the FIRE study.

### Type A facilitation

Type A facilitation was improvement-based, similar to approaches used in primary care in Canada, the UK and the US [14–16]. Evidence from primary care and community health settings indicates that this type of facilitation can enhance the uptake of clinical guidelines [17] and improve health outcomes [18]. Type A facilitation was designed as a 12 month intervention, focused on enabling teams to implement evidence-based care through methods such as audit and feedback and Plan-Do-Study-Act cycles [19]. It promoted a pragmatic, goal-focused approach to implementation [20, 21]. Preparing IFs involved equipping them with skills and knowledge that they could apply within their own teams. This included undertaking an initial assessment of the context and applying audit and improvement methods to work towards locally agreed, evidence-informed goals. The facilitator’s role was to support goal achievement and the process of getting there, for example, by being alert and responsive to group process and contextual issues that could act as barriers to implementation.

### Type B facilitation

Type B facilitation was a 24-month intervention focusing on a practitioner inquiry approach to enable collaborative, inclusive and participative engagement of individuals and teams in the implementation, evaluation and diffusion of research evidence into practice. Type B facilitation explicitly uses critical social science concepts (e.g. consciousness-raising, problematisation, self-reflection and critique) [22, 23], as well as concepts from the new worldview of critical creativity [24], on the basis that the development of individual practitioners, cultures and contexts will result in sustainable change. Action arises because of a desire by individuals or groups to redress observed contradictions, oppressions or domination, rather than action resulting from power or coercion. The intention is to contribute to emancipation—to encourage new ways of thinking and acting. Critical creativity extends the principles of critical social science with a focus on helping practitioners to creatively explore conditions where everyone can flourish.

Like type A, type B facilitation is concerned with change and innovation, but is also explicitly concerned with individual and team learning and effectiveness, leadership and evidence use and development to transform workplace contexts and cultures of care. A realist synthesis of this approach demonstrated the key methods involved and associated outcomes [25].

## Methods

### Setting

Detailed study methods, including site and participant recruitment have been reported elsewhere [1]. Eight sites (two per country in England, Ireland, The Netherlands and Sweden) were randomly allocated to type A facilitation and eight to type B. There were also eight control sites, not discussed further in this paper.

### Participants

**Fig. 2:**
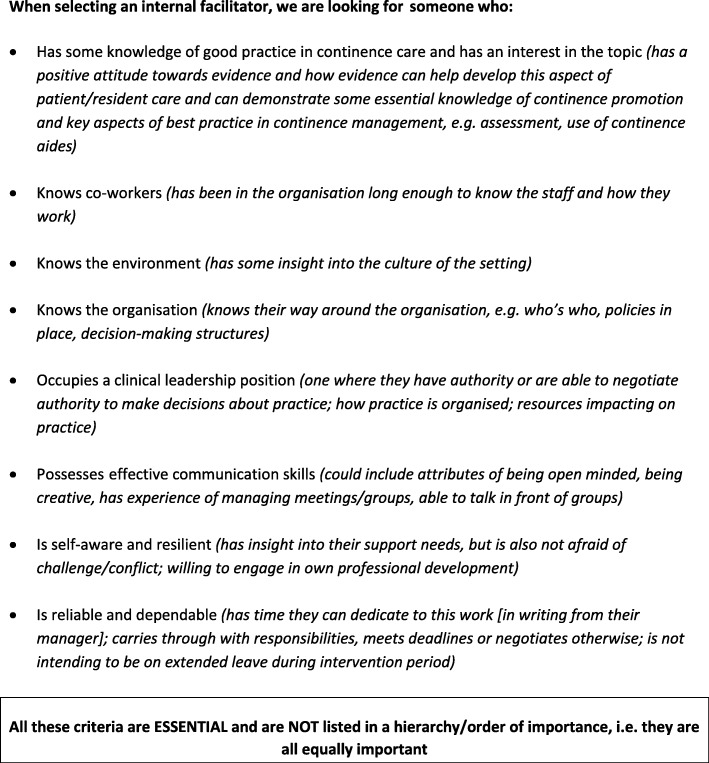
Criteria provided to study sites to guide the selection of internal facilitators (IFs)

Participants were type A and B IFs and the EFs who worked with them. IFs were identified by managers at participating sites, who were asked to invite registered nurses with pre-specified traits, skills and qualities to take on the role (see Fig. 2). Both type A and B IFs were prepared and supported by two EFs (type A: GH and ALK, type B: BMc and AT). To manage the risk associated with an IF leaving during the study, it was recommended that a second nurse be identified as a “buddy.” This was a colleague who could take over if the original IF was unable to continue in the role.

### Designing and delivering the interventions

**Fig. 3:**
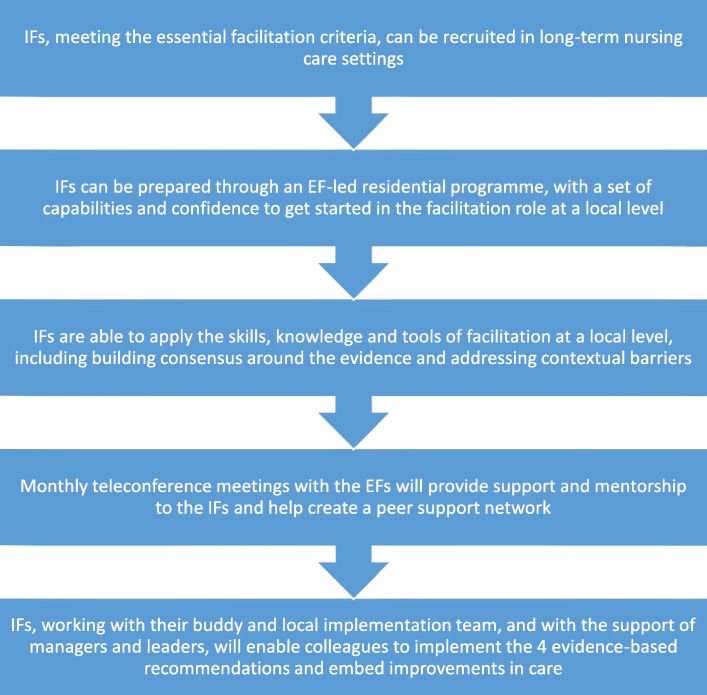
Logic pathway of facilitation intervention

Both interventions were manualised into a logic pathway for the purpose of maintaining and monitoring fidelity during the trial. This pathway reflected similar processes within type A and B facilitation, but with differences in focus, intensity and duration (see Fig. 3 and Table 1). In order to fit with the rationalist paradigm of the cluster randomised controlled trial, the ‘dose’ of facilitation was standardised, as this was a major point of distinction between the two facilitation types, underpinned by a theoretical assumption that the more emancipatory approach of facilitation (type B) would take longer to establish, but could ultimately produce more far-reaching and impactful outcomes. This reflected an original objective of the FIRE study, which was to determine if there was a ‘good enough’ model of facilitation.

**Table 1:** Type A and type B facilitation interventions

Component part of intervention	Type A specific content and focus	Type B specific content and focus
1. IFs, meeting the essential facilitation criteria, can be recruited in long-term nursing care settings	Same criteria for both	
2. IFs can be prepared, through an EF-led residential programme, with a set of capabilities to get started in the facilitation role at a local level	3-day preparation focused on improvement tools and methods, audit and feedback, stakeholder mapping and context assessment and skills in facilitating change.	5-day preparation focused on agreeing ethical processes, stakeholder analysis and engagement in development and inquiry, person-centredness, values clarification, developing a shared vision, workplace culture analysis, developing shared ownership, reflective, active learning, high challenge/high support, 360^o^ feedback, patient/staff stories, observation of care, process and outcome evaluation, facilitation of transitions and use of creative imagination and expression.
3. IFs are able to apply skills, knowledge and tools of facilitation at a local level including building consensus around the evidence and addressing contextual barriers/issues	Establishment of agreed goals for implementation; audit tool and structured implementation plan for the 12-month period IFs set up a local implementation team and work on the activities agreed at the residential programme (e.g. stakeholder engagement, baseline audit, action cycles, etc.).	Exploring the inter-relationship between getting evidence into practice, developing practice, context, culture, evaluation and skilled facilitation, through learning how to engage in co-learning activities with key stakeholders in the organisation in order to build capacity for the delivery of effective evidence-based and person-centred care. IFs set up a local practice development group with whom they engage in co-learning activities experienced during the residential programme (e.g. values clarification, developing a shared vision for evidence-based and person-centred continence care, developing practice, stakeholder engagement and participatory evaluation).
4. Monthly teleconference meetings with the EFs will provide support and mentorship to the IFs and help create a peer support network	12 structured 1 -h meetings based around the agreed implementation plan. Minutes circulated after meeting with EFs reflections for discussion at next meeting.	16 structured 3-h facilitated conversations based on the learning needs of the participants as they progressed their implementation work. A narrative of the conversation was recorded and circulated to participants afterwards. Actions to progress implementation activities were noted/highlighted.
5. IFs, working with their buddy and local implementation team, and with the support of managers and leaders, will enable colleagues to implement the four evidence-based recommendations and embed improvements in care	IFs and local colleagues work systematically through the agreed 12-month implementation plan to audit, implement improvement and re-audit practice against the four guideline recommendations.	IFs, their buddy and the local practice development group systematically work through the stages of implementation and practice development relevant to their local context, informed by co-learning, critical reflection and ongoing participatory evaluation of culture and context.

Once recruited, IFs participated in a residential facilitator development programme (May 2010), led by the relevant pair of EFs. This was delivered face-to-face in a central Netherlands location. The type A programme lasted 3 days and the type B programme 5 days. The outline content for each programme is summarised in Table 2. Following the residential, monthly IF-EF teleconference calls occurred. Type A teleconference support lasted for 12 months (12 teleconferences), whilst in type B, the duration of support was 24 months (16 teleconferences). Accordingly, resourcing of IFs and EFs differed as did the specific activities undertaken, reflecting the different intensity and approach of the interventions (see Table 3). At the end of each intervention, a 24-h closing meeting was held over 2 days. This was an opportunity for the IFs and EFs to reflect on their experiences, progress made, difficulties encountered and suggestions for how the intervention could have been improved.

**Table 2:** Summary of content in type A and type B facilitator development programmes

Day	Type A (3 days)	Type B (5 days)
1	• Introduction and overview • What we know about getting evidence into practice • The evidence for managing urinary incontinence • Implementing guideline recommendations within a local context • Reflection and evaluation	• Understanding about the vision, purpose, structure, facilitation and learning processes of Type B facilitation programme • Use of research evidence in practice, the nature of context (including workplace culture, type of leadership, evaluation) and facilitation and that these things are related • Clarity about nature of the intervention and support from a subject (continence) specialist
2	• Review of day 1 • The knowledge translation toolkit: ο The facilitator role ο Agreeing aims and planning for implementation ο Auditing practice ο Acting on the results of audit • Reflection and evaluation	• Gaining a grasp of the FIRE evaluation framework and the IF role within it • Contextualisation of FIRE evaluation framework in the IFs own setting • Understanding of different types of data and stakeholder engagement for different purposes • Appreciation of how evaluation increases the potential for collaboration, inclusion and participation (CIP)
3	• Review of day 2 • Developing and presenting individual implementation plans • Agreeing the next steps and the time-plan for the 12 months of the intervention • Final reflections and evaluation	• Identification of the skills, tools and resources that are relevant to the IFs context • Understanding how to acquire skills, tools and resources in the IFs context to facilitate implementation of evidence in their setting • Development of targeted use of different approaches • Appreciation of the need to create the conditions for transformation • Recognition of the need to nurture self as a facilitator as facilitation is a long-term activity and change can be slow • Development of strategies to deal with turbulence in the IFs context
4		• Hearing, seeing and embracing the patterns of working collectively and not getting distracted • Translate the role of the facilitator into enabling of different forms of engagement among teams and organisations • Explore tools for meaningful engagement and collective meaningful action • Identify essential processes for enabling engagement and the sustaining of culture change
5		• Be able to articulate the language in the facilitation journey and how this language will be translated into practice in the IFs context • Be equipped with the necessary knowledge and skills to commence the journey of facilitating evidence-informed continence care into practice • Understand how to make use of the tools and processes experienced during this course in the IFs own practice context and be able to adjust their use according to context • Identify what the first steps are and what the IF ‘will do on Monday morning’ to get started with facilitating the guidelines into practice

**Table 3:** Comparison of facilitation ‘dose’ and activities undertaken by type

	Type A facilitation	Type B facilitation
Intervention period	12 months	24 months
Internal facilitator:		
Initial development programme	3 days	5 days
Teleconference meetings	12	16
Funded time to undertake the role	19 days (3 days, development programme; 6 days, teleconferences and personal study; 10 days, implementation and evaluation of guideline recommendation)	43 days (5 days, development programme; 18 days, teleconferences and personal study; 20 days, implementation and evaluation of guideline recommendation)
External facilitator:		
Funded time to undertake the role	16 days per EF	31 days per EF
Type of activities undertaken	• Agreeing improvement goals • Setting up a local project group • Awareness raising, e.g. posters about the project • Audits of continence practice • Development of new documentation, e.g. continence assessment forms and care plan • Supporting staff to complete documentation	• Forming a project group of stakeholders • Values clarification exercise • Self-administered leadership questionnaire • 360° feedback from colleagues • Asking staff to complete Context Assessment Index • Provision of person-centred care presentations to staff • Interviewing residents with urinary continence • Using stakeholder group to identify priorities, agree actions and evaluate progress • Reviewing practice, revising policies and documentation

The EF role was largely separate from the running of the trial. In three countries (England, Sweden and the Netherlands), the trial leads were FIRE project team members from the respective countries who had no direct involvement in delivery of the facilitation intervention. The exception was Ireland where an EF (BMc) was also the country/trial lead.

### Data collection

Qualitative data sources included written notes of monthly teleconference meetings and the closing events and synthesised accounts of interviews with IFs by independent research fellows (RFs; one per country) at 6 monthly intervals. During the teleconference meetings, one EF took on the lead facilitator role and the second EF captured notes of the discussion, which were then shared with the IFs. A similar process occurred at the closing meetings. IF interviews occurred 6, 12, 18 and 24 months after the start of the intervention. These were conducted by the country-level RFs, using a semi-structured interview guide to collect data on the residential programme, progress of implementing guideline recommendations and barriers and enablers influencing implementation. Summary notes of interviews were written by RFs, and those from Swedish and Dutch sites were translated to inform analysis. During data analysis meetings, RFs worked collectively to synthesise data relating to facilitation, as part of the process evaluation [3]. These data summaries were used to inform the analysis for the current paper.

### Data analysis

Data were initially analysed by an independent researcher (EL) who was not involved in data collection or delivering the interventions. Data from each site were collated and checked for accuracy, and any discrepancies were clarified with the EFs. Summary notes from all sources were exported to NVivo 11. Data were broadly mapped to the component elements of the logic pathway (Fig. 3), and data within each grouping were inductively coded. In conducting the data analysis, the purpose was two-fold: firstly to determine how closely facilitation in practice at each site aligned with the logic pathway and secondly to develop an explanatory account of why and how the logic pathway was or was not maintained. A series of teleconference meetings between the independent researcher and the four EFs took place during the data analysis process to discuss and interpret the findings. Notes from the joint analysis meetings were documented to inform our interpretation of the results.

## Results

The results are presented according to the main elements of the logic pathway. Participant quotes are identified in relation to the type of facilitation; feedback from individual IFs is de-identified to maintain anonymity. Key EF reflections relevant to the findings and particularly to the perceived limitations of the logic pathway are also captured.

### Recruitment of IFs meeting essential criteria

Timely identification and long-term retention of IFs were problematic; only 6 of the 16 sites recruited an IF who met the essential criteria and stayed in the role for the intervention period. The requirement to attend the residential programme influenced IF selection at one type A English site, as the preferred facilitator was unable to attend the residential, so an alternate staff member was selected.

One type A site in the Netherlands commenced 6 months after the others due to problems with site recruitment. IFs at 5 sites discontinued in the role due to sick leave (3 type A: England Sweden, Netherlands; 2 type B: Netherlands, Sweden); another 2 discontinued due to leaving the institution (both type B: England and Ireland). IFs at 3 sites did not meet the essential criterion of being in a clinical leadership position: one was a new graduate nurse (Netherlands type B), another a licenced practical nurse (Sweden type B), and a third was an assistant nurse (replacement type A IF: Sweden).

#### EF reflection:

It was clear from the outset that some IFs selected to attend the residential did not fit the ‘ideal type’ facilitator, for example, in terms of personal characteristics, confidence and interpersonal skills. This feedback was provided at the regular FIRE project team meeting; however, given the timetable for the trial and the study resources, there was no option to identify and train an alternative IF. Could the country leads have been better briefed and prepared to negotiate IF recruitment with nursing home managers?

The buddy system had variable success. Buddies became IFs at 4 of the 7 sites where the original facilitators ceased in the role (type A: England and the Netherlands; type B: Ireland and Sweden). New IFs were identified at two sites (type A: Sweden, type B: Netherlands), but at one site (England type B), no replacement was organised when the IF left the organisation.

#### EF reflection:

 From the outset, we recommended that it would be beneficial to have both the IF and buddy attend the face-to-face residential programme, but the study budget was insufficient to support this given the international travel costs involved. This situation was not ideal.

### Preparation of the IFs for the role

Type B IFs at both English sites were unable to attend the residential programme, and one type A Netherlands site commenced 6 months late, so shorter development programmes were organised for the IFs. In response to the turnover of IFs, condensed programmes were organised for replacement IFs at 2 sites (type A: Sweden; type B: Ireland). Replacement facilitators did not receive any formal preparation at 3 sites (type A: England and the Netherlands; type B: Sweden), mainly due to timing and logistical issues.

Facilitator development programmes were delivered in English, so IFs needed to be able to speak and understand the language fluently. Swedish and Dutch interpreters attended the original type A and B programmes to assist with translation. Despite these arrangements, there was consistent feedback from the Swedish and Dutch IFs that aspects of the facilitator development programmes were difficult due to language issues.

The type A residential programme was reported to be beneficial in terms of perceived usefulness of the content, advice and written resources provided and building peer networks. IFs at 3 sites reported that the programme helped to develop a facilitation plan. However, one IF was unable to see what changes could be achieved through facilitation; others reported that they were unclear about the PARIHS framework, which in turn led to uncertainty about role expectations. The 3-day residential was reported by some participants to be too short, with a lot of information provided in the time.

#### EF reflection:

The IF who expressed doubts about facilitation was the only participant at the residential who was also the manager of the nursing home. At the time, we questioned her suitability for the IF role as she did not show any ‘buy in’ for the proposed way of working as a facilitator.

The type B residential programme filled a number of the IFs with enthusiasm and was generally informative and enjoyable. However, not all IFs felt comfortable with the more reflective, emancipatory methods of facilitation—one felt that the approach was not a good fit with her personality and another reported that the proposed facilitation methods would make staff uncomfortable. Two IFs reported a loss of confidence during or after the residential programme. The written resources provided were useful, but both Swedish IFs commented that they were not available in their primary language. Two IFs reported that it would have been useful for the buddy to attend the facilitation programme.

#### EF reflection:

It was evident during the residential programme that working in a second language was challenging for some IFs. Supportive co-learning relationships emerged among the group members to support participants who did not have English as a first language. However, maintaining this level of support after the residential surfaced as a concern. The IFs were returning to their places of work without immediate support being available. In Sweden and the Netherlands, some local facilitation and translation of resources was offered later in the programme, but was only minimally taken up in one setting.

### IF application of facilitation knowledge, skills and tools

Some IFs reported increasing confidence as their knowledge and skills developed, particularly around the topic of continence care.

In part, this may have occurred because additional subject expertise was sought by the EFs. At the type B residential programme, an expert in continence in nursing home care provided an overview of the evidence underpinning the guidelines and facilitated discussion about practical strategies for managing incontinence. Type A EFs also organised a continence expert to join two teleconference meetings when the need for additional knowledge was identified.

#### EF reflection:

Provision of expert input on continence management was not planned into the type A residential programme (unlike type B). In the early teleconferences, it became increasingly apparent that this was something the IFs felt necessary; hence, arrangements were made to invite a continence nurse specialist to two of the teleconferences. This addition to the original type A plan was an example of an adjustment made during the course of delivering the intervention.

A number of IFs reported feeling empowered, having developed their skills and ability to apply facilitation knowledge in practice. However, others commented on a lack of guidance after the residential and a lack of progress, which led to loss of motivation, and inability to identify achievable goals. IFs described ways they had empowered others to improve their performance at 5 of the 16 sites (type A: Ireland, both sites; type B, Ireland, Netherlands and Sweden). Four of these sites retained the original IF in the role throughout the study. Facilitators at other sites did not report empowering staff to make changes; instead, they stepped away from the FIRE project (1 type A, 4 type B) or acted as lone change agents. IFs at 5 sites (3 type A, 2 type B) variously reported assuming sole responsibility for activities such as collecting data, conducting continence assessments, creating and helping complete new documentation. One type A IF reported:

What I have learned is that I may need to step back in the future. That first measurement we [IF and buddy] filled in the forms. We could have let them [staff] do it themselves. In that way their involvement in the project and their motivation to fill in the forms in the future will be higher. However, it was very busy at the time at the ward, so probably nothing would have happened at all [if we had stepped back].

#### EF reflection:

Why did some IFs choose to work alone and not involve other colleagues? This could be because they felt more comfortable with a more project or task-focused approach; in other words, ‘doing for others’ rather than ‘enabling others’.

As expected, there was a difference in the strategies employed by type A and B facilitators. Type A IFs tended to report using systematic processes, such as auditing records (*n* = 4), goal setting, assessing progress and reassessing goals (*n* = 5) and changing paperwork related to continence assessment and management (*n* = 6). By contrast, the most commonly reported strategies by type B IFs included workshops to identify team values and culture (*n* = 5), and the use of creative approaches with staff to engender enthusiasm, track progress and clarify team values (*n* = 4). Three type B IFs also reported changing paperwork for continence assessment or management.

At the majority of sites (type A and B), IFs strove to increase awareness about the FIRE project, by organising meetings, creating posters and fliers and conversing informally with staff. Both types of facilitators reported that data were collected on an ongoing basis from a number of sources (including audit, patient interviews, staff questionnaires, staff stories, scribble boards and informal observations of practice) and that data were used to develop or refine strategies to improve continence care.

Getting teams ‘on board’ was important—type A and B IFs reported being flexible with plans especially in relation to the time required for changes to occur. Progress was often slower than anticipated and both type A and B facilitators employed a deliberate strategy of allowing time for incremental changes and staff acclimatisation. A type B IF explained:

I am pacing myself more now… [There is a] lot of change going on…due to new inspection processes. I have to make sure I don’t overburden people.

EFs similarly recognised issues relating to the timing and pace of planned activities. For example, at the type A residential, the IFs were introduced to an online audit system to input, collate and feedback local audit data, according to a schedule agreed by the EFs and IFs. However, difficulties arose related to the IFs’ ability to use the online system, limited computer access and skills. This slowed down the planned audit process, such that none of the 8 sites reached the point of re-auditing (as originally planned) within the 12 months [26].

#### EF reflection:

We assumed the IFs would have a higher level of knowledge and skills with audit and had not anticipated any difficulties with computer access or use. It soon became clear that the plan we developed with the type A IFs at the residential programme was not going to be realistic for many of them, and it had to be adjusted. A great deal of time at the first few teleconferences was spent trying to sort out the issues with audit, which was difficult as there were 10 or more people on the call. Some IFs ideally needed direct, in-person support to develop their skills and confidence in undertaking the audits. This was not an adaptation deemed feasible, as the EFs were geographically distant, or appropriate, as it would significantly change the ‘dose’ of facilitation.

Despite a large number of reported strategies to improve continence care, these were not applied consistently at different sites or always to good effect. IFs at every site reported that time and conflicting duties were barriers, as they all had substantive roles within the nursing homes. Although funding was provided to allow allocated time for the IF role, protecting this time was not always achieved.

The IF [did not] negotiate with the management to secure protected time, to seek the establishment of resources…This resulted in the IF having to do the work at home and to use her own personal equipment and to do the work on her own time off [research fellow notes relating to Type B IF]

#### EF reflection:

Was the IF’s inability to negotiate protected time linked to the recruitment issue? The recruitment criteria included clinical leadership in some capacity and practice expertise (Fig. 2); thus, the EFs did not focus on the development of such skills in the residential programme and had to respond to issues in the teleconference meetings.

### Mentorship and support through monthly teleconferences

Attendance at teleconferences was variable; IFs from different sites attended between none to all of the scheduled teleconferences. Both type A and B IFs reported that they also contacted the EFs via phone or email if they had queries between teleconferences. Attendance appeared to correlate positively with the ability to apply facilitation skills; 4 of the 5 IFs who reported empowering staff to make changes attended all scheduled teleconferences.

The format of teleconferences allowed for peer support from other IFs, which was seen as beneficial. However, virtual meetings also presented difficulties; facilitators from 7 of the 16 sites reported problems with the technology and IFs from the Netherlands and Sweden expressed language-related problems. Four participants specifically reported that face-to-face meetings would have been better than virtual meetings. As a minimum, it was agreed that a mid-point face-to-face meeting would have been beneficial.

#### EF reflection:

The more engaged IFs were more likely to enact the role as originally envisaged. The issue was what to do about those IFs who did not fully engage or effectively withdrew. Could we have worked more closely with the country leads to encourage them to keep going? The lack of more experienced support and mentorship at a local level was a recurring problem and highlights the central importance of the relational aspects of facilitation.

### IF development and enactment of facilitation

Building an implementation project team was an integral part of successful facilitation, but was achieved at less than half the sites (3 type A, 3 type B). IFs who were able to build effective teams described working with buddies to strategically select people to be involved, including healthcare and management staff from within, and external to, the organisation. Facilitators of successful teams also reported enhancing teamwork by working closely with different parties, communicating regularly and meeting frequently. While some IFs discussed informing residents or families about the project, no site reported including residents or families in the implementation team.

IFs who did not build successful teams reported explaining to, but not involving nurses in implementation (2 sites), or not organising a buddy (4 sites). Three IFs reported working on their own rather than together with other team members.

I keep on struggling with time and motivation of my stakeholders… [There is] no change in the view that people see the project as “[IF’s] project”… They are not ready to take it on and do it well. [Type B IF]

Facilitators from 4 sites reported resistance or a lack of support from staff (for example, due to strong personalities or a lack of priority attached to improving continence), and a further 4 sites reported that management and workplace issues were a major barrier to the facilitation project.

[Staff] never attend any of my workshops so I find it hard to get an opportunity to speak with them. When I visit the wards they find some excuse to disappear. They can often intimidate staff who are very open to change. [Type B IF].

#### EF reflection:

This was typical of the situations where direct input and role modelling from a more experienced facilitator would have been helpful.

### Summary of findings

A number of key issues emerge from the findings. Firstly, it is clear that in reality, the planned interventions did not work according to the documented logic pathway. This reinforces the process evaluation findings, which highlighted mechanisms relating to alignment and fit of the facilitation type to the individual and their organisational context, and subsequent engagement and enactment of the role [3]. Issues and difficulties were encountered with each element of the logic pathway and at critical juncture points such as immediately following the residential programmes. It is also apparent that problems were cumulative, such that if the facilitation intervention started with an inappropriate or ineffective person in the role, then subsequent problems and barriers arose in terms of enacting the role effectively. This included not engaging fully in the teleconference meetings and, in some cases, not contributing to the study (without formal withdrawal). EFs faced difficulties balancing the logic pathway with a more real-world approach to facilitation, which involves working in a fluid and dynamic way. As the reflections illustrate, the EFs were acutely aware of difficulties as the project progressed. These were fed back and discussed at FIRE project team meetings. There was an agreement over some relatively minor adjustments to the delivery of the facilitation interventions, but not to make changes that could affect the dose or intensity of type A or B facilitation, as this was seen to compromise the integrity of the trial.

## Discussion

We return to the questions that framed the paper to structure the discussion: Why did the facilitation interventions, as articulated in the logic pathway, prove difficult to implement in practice? What issues arose in relation to balancing fidelity and adaptation? What lessons were learned that could be beneficial to inform similar research in the future?

### Applying the facilitation interventions within a standardised logic pathway

Challenges in applying the logic pathway for the facilitation interventions in practice related to the study methodology and design, the nature of the intervention being evaluated, and the logistics of a complex, multi-national study in the particularly challenging context of nursing home care. In terms of IF recruitment and selection, the data clearly demonstrate the importance of having the right person in the facilitator role and paying attention to the fit and alignment of the facilitation approach (for example, goal-focused improvement or practitioner-led enquiry) with the individual and organisational characteristics. However, EFs did not have a direct role in the selection of IFs, other than identifying the key selection criteria (Fig. 2). Nor was it possible to address issues of fit and alignment a priori given the randomisation process that was part of the study design. Sometimes, despite the best efforts of sites, there were no staff available that met all the selection criteria. However, more active engagement between the EFs and the country leads responsible for liaising with sites to identify IFs could have helped to ameliorate some of the problems encountered.

### Issues relating to study design

This highlights the tensions inherent in evaluating an intervention that is by nature fluid and iterative within the methodology of an RCT. The research environment imposed a very different set of conditions to the natural, real-world delivery of a facilitation intervention. The logic pathway was primarily developed to address issues of fidelity within the trial, with a particular emphasis on standardising the dose and intensity of facilitation provided in both interventions. From an EF perspective, this imposed limitations in terms of the flexibility that was possible as issues arose during intervention, as illustrated in the findings.

The international scope of the study added another layer of complexity, for example, in terms of coordinating site and IF recruitment so that IFs were able to attend the initial residential programme and making provisions for IFs who had English as a second language. These and other logistical issues were a feature throughout the study and made the IF role in Sweden and the Netherlands especially challenging, particularly if compounded by problems relating to fit and alignment of the facilitation approach. Issues related to staff turnover presented an additional challenge and one that other studies of interventions to change practice in a nursing home context have reported, where workload is high and numbers of registered nursing staff are typically low [27]. This makes the nursing home setting a particularly difficult one in which to implement change and improvement.

### Potential solutions

Strategies that could potentially have been useful include formalising the buddy role into a co-facilitation role and having two IFs per home. Given the geographical distance between sites and between the EFs and IFs, the EFs did not have an opportunity to make site visits, to meet with staff and managers and get a sense of the context in which the IFs were working. Furthermore, there was no face-to-face contact between the IFs and the EFs from the start to end of the intervention. This is different to how a typical EF-IF model would operate, where part of the mentoring relationship would involve direct contact. Having a more experienced facilitator available within individual countries could have provided a valuable bridge between the EF and the IFs and also addressed language barriers where they arose [28]. A short closing event at the end of each programme was negotiated by the EFs at the request of remaining IFs who wanted to share and celebrate their successes and the difficulties they had overcome. But, at the least, building in a mid-point face-to-face meeting would have been beneficial and helped to maintain engagement with the study.

Another factor influencing engagement was the level of managerial support. The EFs had no contact with the managers in the care setting, either at an operational or strategic level. The IF-manager relationship emerged as an important finding [29] and is an area where the EFs could usefully have made some input, for example, through inviting managers to some of the teleconference meetings or having separate information and discussion sessions scheduled with managers to increase their understanding and sense of engagement with the study. This is supported by other implementation studies, which demonstrate the significance of the manager’s role in implementing evidence-based practice in nursing home settings [30, 31].

At one level, the solutions proposed indicate a need to add more to what could already be seen as resource-intensive interventions. However, notwithstanding the fact that facilitation as an implementation strategy involves investment in people and processes, we would argue that it is not necessarily about adding in more. Rather, we suggest it is about doing things differently, particularly within the context of an implementation research study.

### Addressing issues of fidelity and adaptation

Turning to the fidelity-adaptation question, the PARIHS framework emphasises that implementation is multi-faceted and nonlinear. Our experience in FIRE mirrors other studies that have started with a prior theory that recognises implementation as complex, but faces challenges handling complexity in a research context [32]. For both type A and B interventions, progress with implementation was generally less than anticipated and a number of barriers were encountered. In some cases, the EFs took action to address these obstacles by making adaptations, for example, providing expert continence input and adjusting the audit schedule in the type A intervention and revisiting team values and working with the IFs to engage the buddy in type B sites. However, more substantive adaptation or tailoring of the interventions, such as varying the amount and level of support provided at an individual site level in response to specific difficulties that IFs encountered was not undertaken in an attempt to maintain fidelity to the ‘dose’ of facilitation specified in the logic pathway. This was compounded by the restrictions inherent in running a multi-site European trial; for example, the practicalities of increasing face-to-face contact time between EFs and IFs. Overall, this created a source of tension for EFs as it required a way of working that did not mirror how the role functions in the real world.

The fidelity-adaptation debate is one that is increasingly recognised within complex intervention and implementation research studies where context is an important mediating factor [4, 33]. One approach put forward is to identify the core and peripheral (adaptable) components of the intervention to provide clarity around which elements of an intervention can and cannot be subjected to tailoring [34]. In our conceptualisation of fidelity within FIRE, dose represented a core component of the facilitation interventions. An alternative approach would have been to focus fidelity on the intended mechanisms of action, as opposed to the component parts of the intervention. This mechanism-focused perspective on fidelity is one that has been more widely adopted in the field of health promotion and prevention, where interventions subject to evaluation are similarly complex and emergent [35, 36]. Taking this approach, intervention integrity is defined functionally in relation to fit with the theory or principles underpinning the intervention.

Applying this thinking to facilitation, the emphasis would be less on the dose (e.g. a 3- or 5-day residential development programme; 12 or 16 virtual teleconferences) and more on the intended function or purpose of the component. For example, in the case of the initial development programme, the function would be to build skills and knowledge specific to type A or B facilitation. Similarly, if an intended mechanism was to develop confidence in facilitation within the care setting, the focus would not be on how much time was allowed to achieve this; rather, the dose of time, and detailed activities undertaken, would be flexible to enable tailoring to circumstances at a local level, including, for example, specific strategies to involve and engage nursing home leaders and managers. Such an approach clearly poses logistical challenges in the context of managing an externally funded research study with time and resource constraints. As others have commented, “There is a tension between fidelity and adaptation that cannot be resolved easily or simply” [37] (p.2); however, we believe there was a need to achieve a better balance between these two concepts within the FIRE study.

### Reflections and lessons learned

Reflecting on the practical and methodological challenges that we encountered developing, delivering and supporting the facilitation interventions with the FIRE study—and with the benefit of hindsight—what have we learned and what are the things we would do differently if starting out again?

One of the significant issues highlighted from our analysis is the fundamental importance of the relational aspects of facilitation and the need for individuals in a facilitator role to be well supported and mentored. The findings have also led us to reflect on the distinction made between type A and B facilitation. In practice, this distinction could be seen as an arbitrary one as there is a need to adopt the right methods for the right person at the right time and in the right context. This suggests that more blended approaches to facilitation are required, focused around core relational elements. This concurs with recent analyses which relate the mechanisms of action of facilitation to higher-order learning, by way of making connections, dialogue and sense-making [10, 38].

We cannot provide a definitive answer as to whether facilitation was the most appropriate implementation intervention to adopt. As many other implementation studies have shown, there are no easy solutions to changing practice in challenging contexts and there is much still to learn about how best to develop, deliver and research tailored implementation strategies. The findings from this study, coupled with the process evaluation from FIRE, provide us with a conceptual platform for further investigation.

**Fig. 4:**
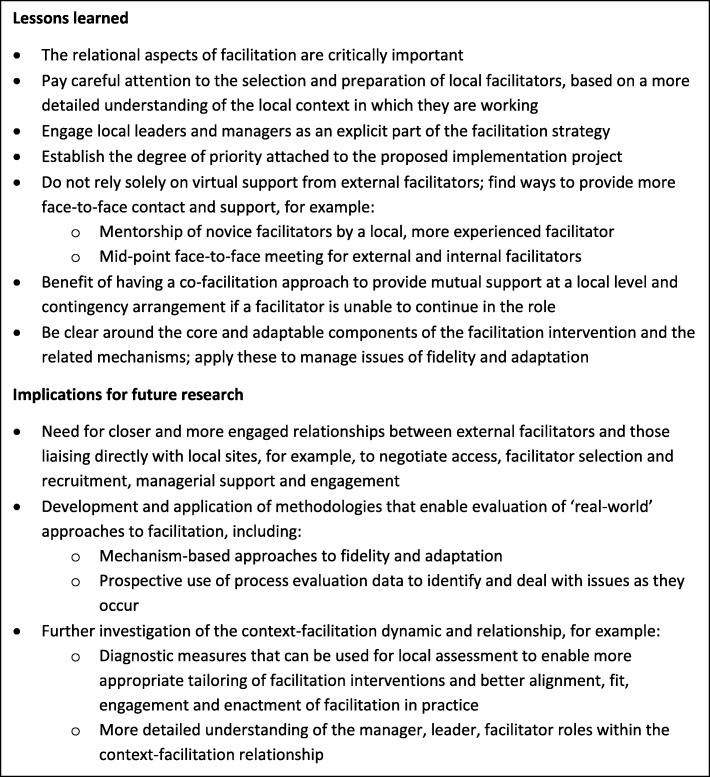
Lessons learned and implications for future research

Figure 4 provides a summary of our reflections on the key lessons learned and implications for future research.

## Conclusions

Evaluating an intervention such as facilitation that is inherently fluid and dynamic within the methodology of an RCT is complex and challenging, particularly in terms of managing the issue of fidelity versus adaptation. In future studies of this nature, we would suggest a theoretical approach to fidelity, with a focus on mechanisms, informed by more prospective use of process evaluation data. At a practical level, relational aspects of facilitation are critically important. It is essential to recruit and retain individuals with the appropriate set of skills and characteristics, explicit support from managerial leaders and accessible mentorship from more experienced facilitators at a local level. Future research to examine the context-facilitation dynamic would help add to the knowledge base on how facilitation approaches can most effectively support implementation and the implementation research agenda in healthcare.
